# Xpert MTB/RIF Ultra on contaminated liquid cultures for tuberculosis and rifampicin-resistance detection: a diagnostic accuracy evaluation

**DOI:** 10.1016/S2666-5247(23)00169-6

**Published:** 2023-09-19

**Authors:** Yonas T Ghebrekristos, Natalie Beylis, Chad M Centner, Rouxjeane Venter, Brigitta Derendinger, Happy Tshivhula, Selisha Naidoo, Rencia Alberts, Bronwyn Prins, Anitta Tokota, Tania Dolby, Florian Marx, Shaheed V Omar, Robin Warren, Grant Theron

**Affiliations:** DSI-NRF Centre of Excellence for Biomedical Tuberculosis Research, South African Medical Research Council Centre for Tuberculosis Research, Division of Molecular Biology and Human Genetics, Faculty of Medicine and Health Sciences (Y T Ghebrekristos MSc, R Venter PhD, B Derendinger BSc, H Tshivhula PhD, R Alberts BSc, Prof R Warren PhD, Prof G Theron PhD), Desmond Tutu TB Centre, Department of Paediatrics and Child Health, Faculty of Health Sciences (F Marx PhD), and DSI-NRF South African Centre of Excellence in Epidemiological Modelling and Analysis (F Marx), Stellenbosch University, Cape Town, South Africa; National Health Laboratory Service, Medical Microbiology, Groote Schuur Hospital, Cape Town, South Africa (Y T Ghebrekristos, C M Centner MSc); National Health Laboratory Service, Greenpoint Tuberculosis Laboratory, Cape Town, South Africa (Y T Ghebrekristos, N Beylis MBBCh, B Prins ND, A Tokota ND, T Dolby ND); Division of Medical Microbiology, Department of Pathology (N Beylis, C M Centner) and Institute of Infectious Disease and Molecular Medicine, Faculty of Health Sciences (S Naidoo MSc), University of Cape Town, Cape Town, South Africa; Division of Infectious Disease and Tropical Medicine, Center for Infectious Diseases, Heidelberg University Hospital, Heidelberg, Germany (F Marx); Centre for Tuberculosis, National TB Reference Laboratory, National Institute for Communicable Diseases a division of the National Health Laboratory Service, Johannesburg, South Africa (S V Omar PhD); Department of Medical Microbiology, Faculty of Health Sciences, University of Pretoria, Pretoria, South Africa (S V Omar)

## Abstract

**Background:**

Xpert MTB/RIF Ultra (Ultra) is a widely used rapid front-line tuberculosis and rifampicin-susceptibility testing. Mycobacterium Growth Indicator Tube (MGIT) 960 liquid culture is used as an adjunct but is vulnerable to contamination. We aimed to assess whether Ultra can be used on to-be-discarded contaminated cultures.

**Methods:**

We stored contaminated MGIT960 tubes (growth-positive, acid-fast bacilli [AFB]-negative) originally inoculated at a high-volume laboratory in Cape Town, South Africa, to diagnose patients with presumptive pulmonary tuberculosis. Patients who had no positive tuberculosis results (smear, Ultra, or culture) at contamination detection and had another, later specimen submitted within 3 months of the contaminated specimen were selected. We evaluated the sensitivity and specificity of Ultra on contaminated growth from the first culture for tuberculosis (next-available non-contaminated culture result reference standard) and rifampicin resistance (*vs* MTBDR*plus* on a later isolate). We calculated potential time-to-diagnosis improvements and also evaluated the immunochromatographic MPT64 TBc assay.

**Findings:**

Between June 1 and Aug 31, 2019, 36 684 specimens from 26 929 patients were processed for diagnostic culture. 2402 (7%) cultures from 2186 patients were contaminated. 1068 (49%) of 2186 patients had no other specimen submitted. After 319 exclusions, there were 799 people with at least one repeat specimen submitted; of these, we included in our study 246 patients (31%) with a culture-positive repeat specimen and 429 patients (54%) with a culture-negative repeat specimen. 124 patients (16%) with a culture-contaminated repeat specimen were excluded. When Ultra was done on the initial contaminated growth, sensitivity was 89% (95% CI 84–94) for tuberculosis and 95% (75–100) for rifampicin-resistance detection, and specificity was 95% (90–98) for tuberculosis and 98% (93–100) for rifampicin-resistance detection. If our approach were used the day after contamination detection, the time to tuberculosis detection would improve by a median of 23 days (IQR 13–45) and provide a result in many patients who had none. MPT64 TBc had a sensitivity of 5% (95% CI 0–25).

**Interpretation:**

Ultra on AFB-negative growth from contaminated MGIT960 tubes had high sensitivity and specificity, approximating WHO criteria for sputum test target product performance and exceeding drug susceptibility testing. Our approach could mitigate negative effects of culture contamination, especially when repeat specimens are not submitted.

**Funding:**

The European & Developing Countries Clinical Trials Partnership, National Institutes of Health.

## Introduction

Rapid molecular tests are essential in the fight against tuberculosis. WHO endorsed Xpert MTB/RIF Ultra (Ultra; Cepheid, Sunnyvale, CA, USA) and Truenat (Molbio Diagnostics, Goa, India) as initial tests for all patients with signs and symptoms of tuberculosis due to short turnaround times and low *Mycobacterium tuberculosis* complex (MTBC) limits of detection.^1–4^ One hitherto underappreciated benefit of these tests is that targeted MTBC DNA detection can occur in the presence of contaminating DNA.

Despite limitations, including expense and time to result, mycobacterial culture is frequently performed for initial tuberculosis diagnosis often following a negative smear microscopy or Ultra result. Reasons for still doing culture in the era of molecular diagnostics are multifactorial and setting-dependent but include clinically justified scenarios that involve presumptive tuberculosis patients with a negative Ultra and HIV (or clinical worsening),^4^ symptomatic patients with recent previous tuberculosis where upfront use of molecular, WHO-recommended rapid diagnostic tests should be avoided^5^ (residual DNA from previous episodes causes false-positive Ultra results),^6^ special groups such as children, or to multiply bacilli for drug susceptibility testing.^7,8^ Furthermore, given high rates of tuberculosis in people with risk factors that do not meet the threshold for symptomatic tuberculosis,^9^ such people are increasingly targeted as part of universal testing strategies by tuberculosis control programmes, including in South Africa.^10^ Culture has an important role in these people as they often have an early-stage disease and low numbers of bacilli.^11^

On an important practical note, GeneXpert capacity is itself uneven within tuberculosis programmes and many settings, even where Ultra is included in routine care, still partly rely on culture.^12,13^ Furthermore, as shown during the COVID-19 pandemic, supply chain disruptions can damage Ultra capacity;^14^ forcing programmes to revert to culture. The Mycobacterium Growth Indicator Tube 960 system (MGIT960; Becton Dickinson Diagnostic Systems, Sparks, NV, USA) is the preferred culture method due to sensitivity and automatability.

Before MGIT960 culture, specimens are decontaminated, centrifuged, and resuspended in buffer; an aliquot of which is used for inoculation. Decontamination differentially reduces bacterial culturability (mycobacteria are typically less affected), making contamination less probable. MGIT960 growth is automatically monitored and, after a tube is flagged as growth-positive, an acid-fast stain is done. If acid-fast bacilli (AFBs) are observed, an antigen or molecular test is done to confirm the presence of MTBC bacteria. If growth occurs but no AFBs are observed, that specimen is reported as culture-contaminated and discarded. A MGIT960 contamination rate of 3–8% is generally considered acceptable.^15^ However, high contamination rates, often attributable to low sodium hydroxide (NaOH) concentrations, have been reported: 30% in Zambia,^16^ 24% in Burkina Faso,^17^ 17% in South Africa,^18^ and 15% in Ethiopia.^19^ After contamination, laboratories should issue a request to health workers to resubmit a new specimen for reinvestigation. This consumes resources, creates a potential care cascade gap and delays diagnoses, including of drug-resistance.

The impact of MGIT960 contamination might be mitigated if AFB-negative growth did not signify the end of a specimen’s journey. Ultra is logical to evaluate; it has well established superior sensitivity compared with smear microscopy on respiratory specimens; it determines rifampicin susceptibility; it is largely automated; and it is often underused, despite being scaled-up in many settings.^20^ We evaluated the sensitivity, specificity, and potential effect of Ultra applied to contaminated MGIT960 growth for the detection of tuberculosis and rifampicin susceptibility.

## Methods

### Study design and samples

In this diagnostic accuracy evaluation study, we used specimens processed in the National Health Laboratory Service (NHLS) Green Point Tuberculosis Laboratory in Cape Town (South Africa). At this laboratory, Ultra is used as the first diagnostic test in presumptive tuberculosis patients and is not used for patients on treatment. Culture is done for paediatric and HIV-positive patients with presumptive tuberculosis when the initial Ultra is negative or in people with recent previous tuberculosis (≤2 years). Culture is also done on patients with Ultra-positive and rifampicin-resistant or rifampicin-resistance indeterminate results. Demographics including age, sex, HIV-status, and tuberculosis-status are collected if available.

As part of routine procedures, specimens for culture were processed using the standard N-acetyl-L-cysteine (NALC)–NaOH procedure for decontamination (1% final concentration).^21^ 0·5 mL of the NALC–NaOH-processed specimens are inoculated into a MGIT960 tube supplemented with polymyxin B (400 units per mL), amphotericin B (40 μg/mL), nalidixic acid (160 μg/mL), trimethoprim (40 μg/mL), and azlocillin (40 μg/mL; PANTA, Becton Dickinson Diagnostic Systems, Franklin Lakes, NJ, USA)^21^ and incubated for maximum 35 days. After a tube is automatically flagged as growth-positive by the machine (200 growth units), Ziehl-Neelsen microscopy is done to detect AFBs on unconcentrated growth. MTBC bacteria identification from AFB-positive growth is done by MTBDR*plus* (version 2.0, Hain Lifescience, Nehren, Germany; if drug susceptibility testing is also required) or the immunochromatographic MPT64 TBc assay (TBc, Becton Dickinson, Sparks, NV, USA; if positive by either test, patient reported as culture-positive) according to the respective manufacturer’s instructions. If only non-AFBs are observed the cultures are reported per programmatic policy as “culture contaminated with no further result to follow”.

For this study, contaminated cultures with no AFB from a smear on growth from a respiratory specimen (sputum or tracheal aspirate) were consecutively collected between June 1 and Aug 31, 2019, and stored at 2–8°C. Results of routine tuberculosis investigations (Ultra, MGIT960, MTBDR*plus*, and TBc) on specimens or isolates up to 3 months after initial contamination detection (ie, follow-up period) were extracted (eg, up until Nov 30, 2019, for contamination detected on Aug 31, 2019). For inclusion in diagnostic accuracy analyses, patients were required to have at least one positive or negative culture result from these later specimens. We did not preferentially select patients based on results from a later specimen other than culture. We excluded patients with a known smear-positive, Ultra-positive, or culture-positive result up to 12 months before the initial contamination report (which would suggest the contaminated specimen was submitted for treatment monitoring) and those who had no later culture-positive or culture-negative results (ie, no reference standard information), and we ignored culture results from any specimens submitted either on the same day as the specimen found to be contaminated or while that specimen was still undergoing incubation (ie, we only included repeat culture results when the repeat specimen was submitted after the initial contamination report). If patients had more than one contaminated culture, the earliest was selected for Ultra or TBc. We included contaminated cultures regardless of whether they were initially tested with Ultra, if Ultra was not positive. We collected meta-data on age, sex, HIV-status, and tuberculosis status if programmatically available.

This study received approval from the Human Research Ethics Committee Division of Molecular and Human Genetics, Department of Biomedical Sciences at Stellenbosch University (S20/08/189) and the NHLS Academic Affairs, Research and Quality Assurance (PR2119347). As we used programmatically submitted de-identified remnant material that would be discarded the need for written informed consent was waived.

### Procedures

After eligible contaminated cultures were selected contaminated cultures were separated based on their later culture result and a subset (later-culture positives and later-culture negatives), most of which either had an initial Ultra-negative sputum result or were not tested by Ultra was consecutively selected and processed for Ultra. 6 mL of contaminated culture were centrifuged (3000 × g 15 min) and the supernatant was discarded, leaving approximately 0·7 mL of pellet, which was resuspended in 1·4 mL sample reagent (Cepheid, Sunnyvale, CA, USA). The concentrated contaminated culture was then tested by Ultra according to our standard operating procedure (appendix pp 2–6). This step consumed all contaminated material, so re-decontamination and repeat Ultra were not possible. Ultra on contaminated MGIT960 is an off-label indication unvalidated by the manufacturer. For TBc 100 μL of Ultra-positive (n=20) or Ultra-negative (n=20) contaminated cultures (without concentration) were tested from randomly selected patients (equal numbers of each semiquantitation category; random selection performed using the RAND function of Microsoft Excel version 365).

If at least one subsequent culture was MTBC-positive the patient was designated as definite tuberculosis. If there was no MTBC-confirmed growth and no other MTBC-positive cultures during the follow-up period, the patient was designated as non-tuberculosis. For rifampicin susceptibility, reference standard resistant cases had definite tuberculosis and were MTBDR*plus* rifampicin-resistant on a subsequent isolate; reference standard susceptible cases were definite tuberculosis and MTBDR*plus* rifampicin-susceptible.

### Statistical analysis

Sensitivity and specificity of Ultra and TBc on contaminated cultures for tuberculosis and rifampicin susceptibility were estimated using 2 × 2 tables with 95% CIs (exact binomial method calculated using Excel and analysed across Ultra semiquantitation categories (ie, trace, very low, low, medium, and high).^1^ Using the prtest command in Stata (version 17), we compared Ultra sensitivity and specificity between patients with previous tuberculosis (confirmed on a specimen submitted less than 4 years but more than 1 year before the contaminated specimen) with those with no previous tuberculosis to evaluate whether, as observed with sputum,^6,22^ specificity diminished. Using Excel, we visualised how positive predictive values (PPVs) and negative predictive values (NPVs) change with the frequency of tuberculosis and rifampicin-resistance in people who had another specimen. Tuberculosis frequency was defined as the proportion of individuals with culture-positive specimens in those patients who had another specimen submitted within 3 months of the first contaminated culture and for whom that later specimen was culture-positive or culture-negative. For rifampicin-resistance, frequency was the proportion of patients who had a MTBDR*plus*-rifampicin-resistant specimen among those who had another specimen submitted within 3 months that was culture-positive and MRBDR*plus* tuberculosis-positive. We included sequential contaminated samples until high precision was achieved for sensitivity and specificity (≤5% CI widths on either side of the point estimates).^23^ Sensitivity and specificity changes were evaluated if trace results (ie, the lowest Ultra semiquantitation category) were recategorised as negative or excluded.

We designated patients with a contaminated culture who had no record of any repeat specimen in the follow-up period as lost to follow-up. Diagnostic delay caused by contamination was defined as days between report of the initial contamination result and, if not lost to follow-up, the earliest next-positive result (Ultra, smear, or culture) on a later repeat specimen. If patients had repeat specimen results and none were positive, the earliest culture-negative result date was used. The difference in the initial specimen culture contamination report date (plus 1 day) and the repeat specimen culture result date was defined as the potential improved turnaround times.

### Role of the funding source

The funders of the study had no role in study design, data collection, data analysis, data interpretation, or writing of the report.

## Results

**Figure 1:**
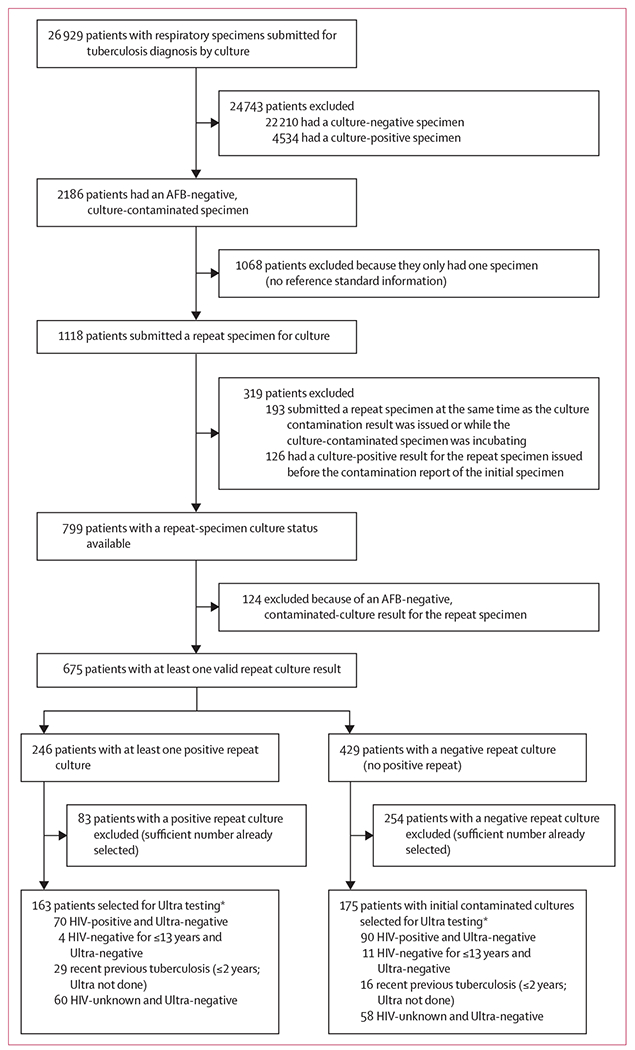
Study profile AFB=acid-fast bacilli. Ultra=GeneXpert MTB/RIF Ultra. *Contaminated cultures were consecutively selected for Ultra based on their later culture result (not all eligible contaminated cultures were tested as detailed in the Methods). If patients had more than one contaminated culture, the earliest-available contaminated culture was selected for Ultra (hence one contaminated culture was tested per patient).

Between June 1, 2019, and Aug 31, 2019, 36 684 specimens from 26 929 unique patients were processed for diagnostic culture (figure 1). 18 936 (70%) of 26 929 patients had one specimen submitted for culture, accounting for 18 936 (52%) of 36 384 specimens. The remaining 7993 (30%) patients had 2 or more specimens submitted, amounting to 17 748 (48%) specimens.

Of all 36 684 specimens, 27 921 (76%; from 22 210 patients) were culture-negative, 6361 (17%; from 4534 patients) were culture-positive, and 2402 (7%; from 2186 patients) were culture-contaminated (without AFBs).

1068 (49%) of 2186 patients with a culture-contaminated specimen had no further specimens submitted (figure 1; these individuals had no differences in available meta-data compared with people with a subsequent specimen; appendix p 7). After exclusions, there were 675 eligible patients with reference standard information. The median time between first culture contamination report and the second specimen culture report date was 42 days (IQR 30–54).

338 patients, 163 with culture-positive repeat specimens and 175 culture-negative repeat specimens, were selected for Ultra testing. Three specimens (1%) from these patients yielded a non-actionable Ultra result (all invalid). Ultra detected tuberculosis in 144 (89% ) of 161 patients with initially contaminated cultures and culture-positive repeat specimens (true positives); ten (7%) of these were in the trace, 12 (8%) in the very low, 37 (26%) in the low, 39 (27%) in the medium, and 46 (32%) in the high Ultra semiquantitation categories, respectively. Of the non-trace categories, 21 (16%) of 133 patients were Ultra rifampicin-resistant. Ultra detected tuberculosis in nine (5%) of 174 patients with contaminated cultures with culture-negative repeat specimens (false positives; seven trace, rifampicin resistance indeterminate, and two non-trace, rifampicin susceptible).

For tuberculosis detection, sensitivity was 89% (95% CI 84–94; 144 of 161) and specificity was 95% (90–98; 165 of 174). For rifampicin-resistance detection, sensitivity was 95% (75–100; 19 of 20) and specificity was 98% (93–100; 100 of 102).

If trace calls were reclassified to negative, sensitivity decreased to 83% (77–89; 134 of 161) and specificity improved to 99% (96–100; 172 of 174).

Previous tuberculosis was more frequent in false-positive than true-positives (six [67%] of nine *vs* 24 [17%] of 144; p<0·0001) and hence specificity reduced in people with previous tuberculosis (67% [12/18], 95% CI 41–87 *vs* 98% [153/156], 94–100 in those with no previous tuberculosis; p<0·0001). This result did not change with different trace recategorisation strategies (appendix p 7).

**Figure 2:**
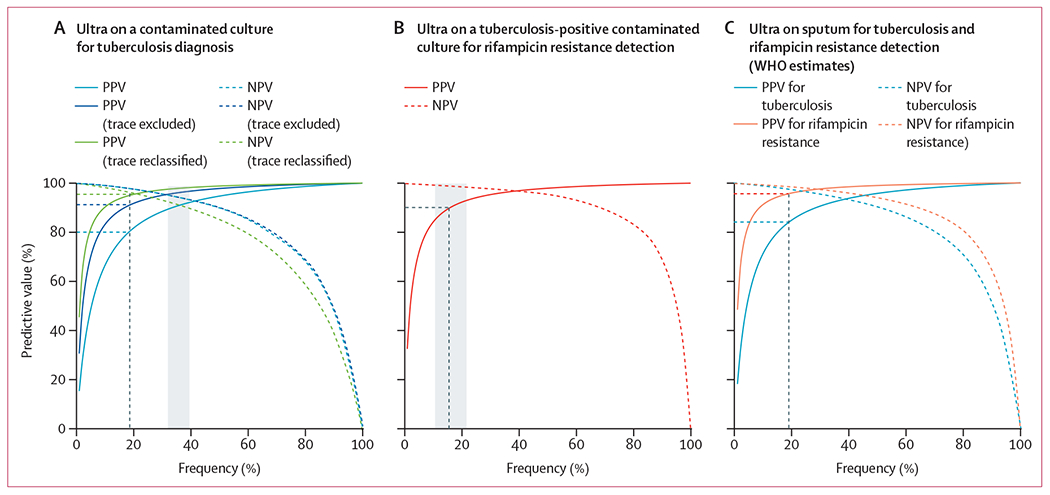
Predictive values of Ultra (A) Predictive value of Ultra on AFB-negative, contaminated MGIT960 growth as a function of frequency of tuberculosis (ie, the proportion of patients with an initial culture-contaminated specimen and a later culture-positive specimen); the grey area indicates the observed frequency (36%, 95% CI 33–40); at a frequency of 18%, half of that observed in this cohort (indicated by the black dashed vertical line), Ultra’s PPV is 80%, increasing to 91% with trace exclusion or 95% with trace reclassification strategies, approximating or exceeding that of Ultra on sputum. The curves for NPV and NPV (trace excluded) cannot be readily distinguished because they are almost identical. (B) Predictive value of Ultra on contaminated growth as a function of frequency of rifampicin-resistance (ie, the proportion of patients with an initial culture-contaminated specimen and a later culture-positive, rifampicin-resistant isolate); the grey area indicates the observed frequency (16%, 95% CI 10–23); at the observed frequency, Ultra’s PPV for rifampicin-resistance is 90%, approximating that of Ultra on sputum. (C) Predictive value of Ultra on sputum according to WHO estimates as a function of the proportion of patients with tuberculosis or rifampicin-resistant tuberculosis;^24^ at a frequency of 19%, Ultra’s PPV is 84% for tuberculosis and 96% for rifampicin resistance. AFB=acid-fast bacillus. NPV=negative predictive value. PPV=positive predictive value. Ultra=Xpert MTB/RIF Ultra.

Among individuals who had an initial contaminated culture tuberculosis frequency was 36% (95% CI 33–40; figure 2A), at which the PPV of Ultra was 91% (90–92) and NPV was 94% (93–94). This result did not differ with different trace recategorisation strategies. In a setting where the frequency in patients initially culture contaminated is approximately half that in our cohort (figure 2A), the PPV would be 80% (78–81) and the NPV would be 98% (97–98); with PPVs increasing to 96% (95–97) and 98% (97–98) and NPVs remaining similar at 94% (93–95) and 91% (91–92) with trace exclusion and reclassification strategies, respectively.

Predictive values as a function of the proportion of culture-contaminated patients with a later submitted tuberculosis-positive culture that was rifampicin-resistant is in figure 2B. Frequency of resistance was 16% (95% CI 10–23), resulting in a PPV of 90% (89–91) and NPV of 99% (99–99).

These predictive values were similar to those for Ultra on sputum for tuberculosis and rifampicin susceptibility estimated using sensitivities and specificities from a systematic review and meta-analysis used by WHO for policy making (figure 2C).^24^

**Figure 3:**
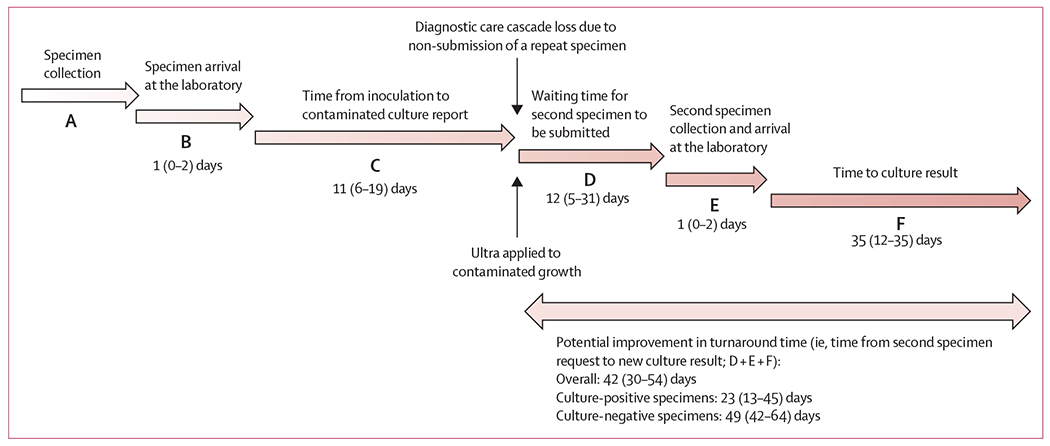
Concept map The concept map shows the timeline from the date of initial specimen collection (A) to when it arrives at the laboratory for processing (B) and when it is reported as contaminated (C). At this point, where indicated by the upper vertical arrow, substantial care cascade loss occurs due to a repeat specimen not being submitted. This loss, and the subsequent delays to await collection of another specimen (if received at all; D), deliver to the laboratory (E), and culture the sample (F) could be minimised if the Ultra on contaminated culture approach were applied (bottom vertical arrow) All day values are median (IQR). Ultra=Xpert MTB/RIF Ultra.

The potential improved turnaround times for diagnosis was a median of 42 days (IQR 30–50) overall, 23 days (13–45) for patients whose repeat culture was positive and 49 days (42–64) for those whose repeat culture was negative (figure 3). For rifampicin susceptibility, potential improvement in turnaround times was 24 days (15–49).

From the 20 randomly selected MGIT960 tubes where Ultra had detected tuberculosis, one was tuberculosis-positive by TBc, resulting in a sensitivity of 5% (95% CI 0–25). The 20 tubes where Ultra did not detect MTBC were all TBc-negative.

## Discussion

This study is the first to describe rescuing a result from contaminated cultures using a WHO-recommended rapid molecular test, Ultra. We found that Ultra on contaminated cultures is comparable to Ultra on sputum for tuberculosis and rifampicin-resistance detection, and that this could reduce delays in diagnosis associated with the need to collect and culture a second specimen or, even more importantly, generate a diagnostic result (with high sensitivity and specificity) where there is none due to non-submission of a repeat specimen. Moreover, we identified many patients receiving multiple cultures simultaneously or in quick succession, which indicates wasteful testing. Together, these findings have implications for improving tuberculosis and drug-resistant tuberculosis diagnosis and can reduce some of the disadvantages of using liquid culture.

Our approach detected nine of ten tuberculosis cases, a sensitivity approximating those previously reported for sputum Ultra.^1,2^ By contrast, TBc performed poorly on AFB-negative contaminated MGIT960 cultures relative to Ultra. Furthermore, the Ultra approach had lower non-actionable result rates (not Ultra-positive or Ultra-negative) than those reported by other studies in our setting,^2^ probably because culture growth, although contaminated, is more homogenous than sputum. We modelled how the predictive value of our approach would change at different rates of definite tuberculosis and rifampicin resistance to offer a framework for different settings to consider rolling out our approach, and showed that these predictive values mirrored those widely accepted for Ultra on sputum. The high tuberculosis (and rifampicin-resistance) frequency in people who were initially culture-contaminated was surprising, but could be confounded by differences in the types of individuals who are likely to have a repeat specimen retrieved (*vs* those who are not) or by tuberculosis-associated perturbations in the respiratory microbiome that increase culture of contaminating organisms,^25^ about neither of which we have information.

We evaluated the effect of different trace handling strategies on sensitivity and specificity but these resulted in small specificity improvements at a cost of missed definite tuberculosis. False-positive results (Ultra-positive on the contaminated culture and subsequently culture-negative) might be due to old MTBC DNA because our approach had slightly diminished specificity in patients with previous tuberculosis (patients who complete treatment can continue to be Xpert-positive for years thereafter);^6,22^ however, this possibility requires further investigation.

For rifampicin resistance, Ultra on contaminated culture had high concordance with MTBDR*plus* on the repeat-culture isolate. The two patients with discordant rifampicin susceptibility results by Ultra (resistant) and MTBDR*plus* (susceptible) could be due to heteroresistance, although Ultra melt curves did not show heteroresistance (these individuals had no record of any other specimens, received the first-line regimen, and died on treatment). For the patient with a rifampicin-susceptible contaminated culture by Ultra and rifampicin-resistant by MTBDR*plus*, three repeat specimens were submitted for culture and MTBDR*plus* (two were MTBDR*plus* susceptible and the last resistant).

While our approach will probably reduce time to diagnosis, its most notable effect is likely to be in the many patients who do not have a repeat specimen submitted. Their tuberculosis diagnoses (and potential rifampicin-resistance) is lost by the system. We also observed over-requesting of cultures, with many simultaneous or repeat cultures in rapid succession in approximately 30% of patients. The combination of loss from the care cascade and algorithm non-compliance indicates an important area for improvement in quality of care and highlights the potential usefulness of refining specimen gatekeeping systems by, for example, denying a culture request if another culture is underway or was recently completed. Our approach could partly mitigate these issues by reducing need to submit another specimen for culture if Ultra salvaged a result from a contaminated culture.

A first limitation of our approach is that Ultra was done on a different specimen to the reference and changes in concentration of bacilli in between samplings could lead to discordance; however, this would favour the reference standard (the index test was done on the early more paucibacillary specimen), meaning our estimates are probably underestimations. Second, it is impossible to exclude the possibility of patients having started treatment between provision of the specimen for culture and a repeat. However, treatment would render patients culture-negative and result in poor specificity—a phenomenon we did not observe. Third, given the pragmatic nature of our study, there was a large degree of missingness (due to no subsequent programmatic culture), but the high missingness demonstrates our approach’s potential value in preventing pre-treatment loss to follow-up. Fourth, our laboratory uses a 35-day MGIT960 incubation period due to limited space, which might have affected the reference standard. Fifth, TBc might have performed better on AFB-positive contaminated growth or if concentrated contaminated growth was tested (as done for Ultra). Sixth, the usefulness of our approach scales with the extent of cultures’ deployment (and contamination rate) and the value of our findings is limited where these are rare, but the added value of our approach will be increased in high-burden settings with higher sample contamination rate than ours (7%). Lastly, our work should be viewed as a proof of concept and should be validated in other settings and using other PCR tests.

In conclusion, Ultra on contaminated cultures is highly accurate to diagnose tuberculosis and rifampicin resistance. As Ultra’s cost approximates that of culture and our approach has many potential advantages (eg, reduced loss from the cascade of care and improved turnaround time), we strongly advocate for laboratories that experience contamination in tuberculosis diagnostic cultures to consider implementing our approach, in addition to maintaining their contamination rate within an acceptable range.

## Supplementary Material

1

## Data Availability

Study data can be accessed on request from the corresponding author without restriction.

## Research in context

### Evidence before this study

Improving the diagnosis of tuberculosis and drug-resistance through strengthening the laboratory care cascade is a public health priority. Despite the scale-up of molecular tests like Xpert MTB/RIF Ultra which has improved diagnostic care, culture is still widely used globally and remains an important tool in some risk groups. However, culture contamination prevents generation of a result and requires collection of another specimen, causing diagnostic delays or drop-outs from the care cascade. We searched PubMed from database inception to June 30, 2022, using the terms “contaminated culture” AND “Xpert” OR “molecular assay” with no language restrictions and identified no studies or information about testing contaminated cultures done for tuberculosis diagnosis using any molecular method. Therefore, the use of Ultra on contaminated cultures, especially if acid-fast bacilli-negative, is unexplored. If performance is high, the negative effect of culture contamination, which is frequent in many settings, could be drastically mitigated as Ultra is a widely available WHO-approved molecular assay.

### Added value of this study

We showed that Ultra on contaminated Mycobacterium Growth Indicator Tube 960 (MGIT960) cultures (which would normally be discarded) can detect tuberculosis with high sensitivity and specificity. This approach also had excellent sensitivity and specificity for rifampicin resistance. Performance levels exceeded those accepted by WHO for Ultra done directly on respiratory specimens. In patients who had another specimen submitted for culture after initial culture contamination, our approach could reduce time to diagnosis. Critically, many patients with contamination had, despite programmatic guidance requiring it, no record of a further attempt to diagnose tuberculosis. In such patients, use of Ultra on contaminated cultures would result in an accurate tuberculosis and rifampicin-resistance result where none would ordinarily occur.

### Implications of all the available evidence

Laboratories should consider evaluating and potentially implementing Ultra on contaminated diagnostic MGIT960 cultures, which would likely improve the diagnosis of tuberculosis and drug-resistance. This approach should not reduce continuous efforts to improve and maintain the quality of culture but, when contamination does inevitably occur, mitigate contamination’s impact on the care cascade.
