# Supplementary material for: Xpert MTB/RIF Ultra on contaminated liquid cultures for tuberculosis and rifampicin-resistance detection: a diagnostic accuracy evaluation
Source: Lancet Microbe. Author manuscript; Available in PMC 2023 Oct 26. (PMC10600950; doi:10.1016/S2666-5247(23)00169-6)
Supplement: 1 [file NIHMS1935676-supplement-1.pdf]

THE LANCET
Microbe

Supplementary appendix

This appendix formed part of the original submission and has been peer reviewed. We post it as supplied by the authors.

Supplement to: Ghebrekristos YT, Beylis N, Centner CM, et al. Xpert MTB/RIF Ultra on contaminated liquid cultures for tuberculosis and rifampicin-resistance detection: a diagnostic accuracy evaluation. *Lancet Microbe* 2023; published online Sept 19. https://doi.org/10.1016/S2666-5247(23)00169-6.

Supplementary Table of contents:

1. SOP for Xpert MTB/RIF Ultra on contaminated MGIT cultures…………………………………2
2. **Table 1.** Comparisons of available meta-data showing no detectable differences between people who had a subsequent specimen submitted for culture and those that did not (sensitivity analysis). Data are % (95% CI) (n/N) or median (IQR). ……………………………………………………7
3. **Table 2.** Sensitivity and specificity of Ultra for MTBC and rifampicin resistance detection on contaminated MGIT960 cultures against the reference standard. Data are n/N (%) with (95% CI)………………………………………………………………………………………….7

Supplementary Methods: **SOP Title**: Xpert MTB/RIF Ultra on Contaminated MGIT tubes

CLINICAL MYCOBACTERIOLOGY AND EPIDEMIOLOGY (CLIME) RESEARCH GROUP

Division of Molecular Biology and Human Genetics
Faculty of Medicine and Health Sciences
Laboratory Standard Operating Procedure

**SOP FOR LAB ACTIVITY_XPERT MTB/RIF ULTRA IN CONTAMINATED MGIT TUBES**	
**SOP #: LP_CLIME_YONAS_001**	**VERSION 1.0**<br>**EFFECTIVE DATE:**<br>**01/05/2021**
**PREPARED BY:**	**APPROVED BY PRINCIPAL INVESTIGATOR:**
Yonas Ghebrekristos<br>PhD Candidate<br>CLIME<br>Email: yonasg@sun.ac.za	Prof Grant Theron<br>Principal Investigator<br>CLIME<br>Email: gtheron@sun.ac.za<br><br>**APPROVED BY QA:**<br>Dr Megan Hendrikse<br>Laboratory Manager<br>CLIME<br>Email: mhendrikse@sun.ac.za

A. Purpose

This standard operating procedure (SOP) describes in detail the steps to follow when processing and performing Xpert MTB/RIF Ultra testing on Mycobacterium Growth Indicator Tube (MGIT) cultures

The workflow is as follows:

Growth detected in MGIT by instrument

Perform AFB smear (Ziehl Neelson or Auramine stain)

- Smear positive then perform standard speciation test
    - speciation test positive then TB
    - speciation test negative then NTM
- Smear negative and contaminating organisms observed then perform Ultra
    - Ultra positive then TB
    - Ultra negative then contaminated

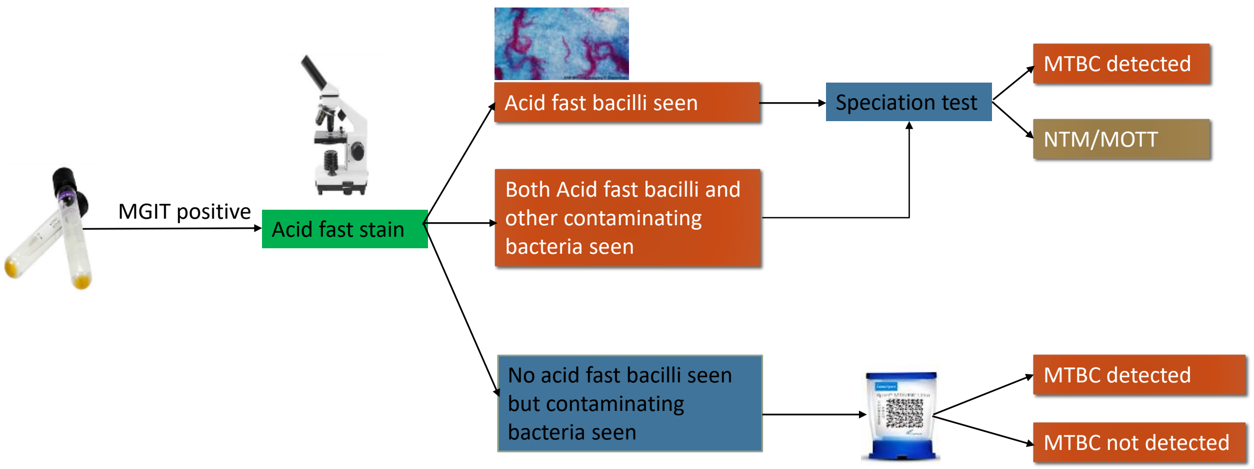

B. Supplies needed

1. Biosafety Cabinet
2. Xpert Ultra kit (Xpert Ultra cartridges, Sample Reagents (SR) and sterile transfer pipettes) Xpert DX system (GeneXpert instrument, computer, software, scanner, printer, and UPS) 5 ml centrifuge tubes

3. 50 ml centrifuge tubes Centrifuge
4. Pasteur pipettes P1000 pipette Filter tips
5. Timer (stopwatch)

Notes:

- Store the Xpert Ultra cartridges and reagents at 2 to 28 °C
- Do not use cartridges or reagents beyond the expiration date
- Do not open cartridge lid until ready to perform the testing

C. Responsibilities

1. All staff working in the Laboratory are responsible for the implementation of this Standard operating procedure.
2. All users of this procedure who do not understand it or are unable to carry it out as described are responsible for seeking advice from their supervisor.
3. It is the responsibility of the lab personnel to follow this SOP as it is written.
4. It is the responsibility of the lab personnel to follow the necessary safety precautions when handling MGIT cultures during this process.

D. Safety

1. Only trained personnel will perform Xpert testing in MGIT tubes
2. Before you start to use the Gene Xpert Dx system, make sure you read the operator manual available in the lab. Using controls, making adjustments, or performing procedures other than those specified in this SOP may result in hazard or injury to person or damage to the equipment.
3. Do not attempt to lift the instrument without proper safety training and assistance. This may damage the instrument and void the warranty.
4. Read and follow the instructions in the product insert of Xpert Ultra cartridge.
5. All processing of clinical specimens should be performed as per the biosafety standards.
6. Treat all MGIT media, including used cartridges, as potentially infectious.
7. Wear protective gloves and laboratory coats when handling specimens and reagents.
8. Wash hands thoroughly with soap and water after handling specimens and test reagents.
9. All benches should be decontaminated after work, or immediately after a spill, with an appropriate Mycobactericidal disinfectant, followed by 70% alcohol.
10. Do not reuse Xpert Ultra cartridges.
11. Dispose of used Xpert Ultra cartridges according to infectious waste material disposal guidelines.

E. Processing and testing.

1. A MGIT tube containing 7 to 8 ml of liquid culture will be tested using the Ultra assay.

2. If the volume is >7ml; 6 ml will be centrifuged at 3000 xg for 15 minutes and supernatant will be discarded into a waste container with appropriate disinfectant. The pellet will be used for further Ultra testing.
3. If the volume is <7 ml; 1 ml the culture will be stored, and the leftover will be centrifuged at 3000 xg for 15 minutes and supernatant will be discarded into a waste container with appropriate disinfectant. The pellet will be used for further Ultra testing.
4. Any leftover culture will be stored at -20°C for further investigation if needed.

F. If Volume of MGIT is ≥ 7 ml:

1. Label one 50/15 ml centrifuge tube with the patient study number, date and specimen type i.e. “MGIT”.
2. Transfer 6 ml of the culture to the 50 ml tube and concentrate by spinning at 3000 xg for 15 minutes at room temperature. Save the rest of the culture in the MGIT tube.
3. Using a sterile Pasteur pipette, remove the supernatant and discard into appropriate disinfectant, leaving a volume of 0.7ml of the supernatant and vortex thoroughly.
4. Add 1.4 ml of Xpert Ultra sample reagent buffer using a Pasteur pipette. The treated specimen can only be used until 4 hours from the the addition of sample reagent.
5. Seal the lid of the 50 ml centrifuge tube tightly and vortex thoroughly for 60 seconds and until no clumps are observed.
6. Incubate for 10 minutes at room temperature
7. Vortex thoroughly for 60 seconds.
8. Incubate the sample at room temperature for an additional 5 minutes
9. Label the Ultra cartridge with the sample study number
10. Mix sample by swirling gently
11. Using a sterile Pasteur pipette, transfer 2 ml of the sample and reagent mixture to the Ultra cartridge, avoiding bubbles
12. Load cartridge into the GeneXpert instrument as per manufacturer’s instructions as soon as possible.

G. If Volume of MGIT is < 7 ml

1. Label a 50 ml centrifuge tube with the patient study number, date and specimen type i.e. “MGIT”
2. Store 1 ml of the MGIT culture in a microcentrifuge tube and transfer the remaining culture to the 50 ml tube
3. Concentrate culture by spinning at 3000 xg for 15 minutes at room temperature

H. Repeating Ultra using unconcentrated MGIT:

1. This is to be used in the event of Ultra test resulting in non-actionable result that would require the Ultra to be performed again.

2. Non-actionable result occurs when the assay run is unsuccessful and is reported as an "invalid, no-results or error" by the GeneXpert instrument. This could be due to system control failure, improper sample preparation, power interruption or failure etc.

A. Add 2 ml of Ultra sample reagent buffer to the 1 ml aliquot of MGIT culture

B. Vortex thoroughly for 60 seconds

C. Incubate at room temperature for 10 minutes

D. Vortex thoroughly for 60 seconds

E. Incubate at room temperature for a further 5 minutes

F. Add 2 ml sample- Xpert reagent mixture into a correctly labelled Ultra cartridge, avoiding bubbles

G. Load cartridge into the Xpert instrument as per manufacturer's instructions

I. Definitions

MGIT	Mycobacterium Growth Indicator Tube	SR	Sample Reagent
MTBC	*Mycobacterium tuberculosis* complex	rpm	Revolutions per minute
SOP	Standard Operating Procedure	BSC	Biosafety cabinet

Supplementary Table:

Table 1. Comparisons of available meta-data showing no detectable differences between people who had a subsequent specimen submitted for culture and those that did not (sensitivity analysis). Data are % (95% CI) (n/N) or median (IQR).

	No later specimen	>=1 later specimen
Known HIV-status	62% (59-65; 659/1068)	66% (63-69; 735/1118)
HIV-positive	80% (77-83; 526/659)	77% (74-80; 569/735)
Male	56% (53-59; 597/1068)	60% (57-63; 670/1118)
Age (years)	37 (26-50)	39 (29-49)
Time-to-positivity (for the initial contaminated culture, days)	9 (4-20)	10 (5-17)

CI = confidence interval, IQR = interquartile range

Table 2. Sensitivity and specificity of Ultra for MTBC and rifampicin resistance detection on contaminated MGIT960 cultures against the reference standard. Data are % (95% CI); (n/N).

Ultra result from the contaminated MGIT960 culture	Sensitivity	Specificity
MTBC detected (any Semi-quantitative)	89% (84 – 94; 144/161)	95% (90 – 98; 165/174)
MTBC Trace excluded	89% (83 – 93; 134/151)	98% (96 – 100; 165/167)
MTBC Trace classified as negative	83% (77 – 89; 134/161)	99% (96 – 100; 172/174)
MTBC detected (any Semi-quantitative) with no previous TB	91% (85 – 95; 120/132)	98% (94 – 100; 153/156)
MTBC detected (any Semi-quantitative) with previous TB	83% (64 – 94; 24/29)	67% (41 – 87; 12/18)
MTBC Trace excluded with no previous TB	90% (83 – 95; 110/122)	100% (98 – 100; 153/153)
MTBC Trace excluded with previous TB	83% (64 – 94; 24/29)	86% (57 – 98; 12/14)
MTBC Trace classified as negative with no previous TB	83% (76 – 89; 110/132)	100% (98 – 100; 156/156)
MTBC Trace classified as negative with previous TB	83% (64 – 94; 24/29)	89% (65 – 99; 16/18)

MTBC = *Mycobacterium tuberculosis* complex, MGIT960 = Mycobacterium Growth Indicator Tube
